# Supplementary material for: Psychosocial work factors and sick leave risk after a terrorist bomb attack: a survey and registry-based longitudinal study of governmental employees in Norway
Source: BMJ Open. 2021 Oct 19;11(10):e052628. doi: 10.1136/bmjopen-2021-052628 (PMC8527151; doi:10.1136/bmjopen-2021-052628)
Supplement: Author's manuscript [file bmjopen-2021-052628.draft_revisions.pdf]

BMJ Open

BMJ Open is committed to open peer review. As part of this commitment we make the peer review history of every article we publish publicly available.

When an article is published we post the peer reviewers' comments and the authors' responses online. We also post the versions of the paper that were used during peer review. These are the versions that the peer review comments apply to.

The versions of the paper that follow are the versions that were submitted during the peer review process. They are not the versions of record or the final published versions. They should not be cited or distributed as the published version of this manuscript.

BMJ Open is an open access journal and the full, final, typeset and author-corrected version of record of the manuscript is available on our site with no access controls, subscription charges or pay-per-view fees (http://bmjopen.bmj.com).

If you have any questions on BMJ Open's open peer review process please email info.bmjopen@bmj.com

BMJ Open

Psychosocial work factors and sick leave risk after a terrorist bomb attack: A prospective study among governmental employees in Norway.

Journal:	*BMJ Open*
Manuscript ID	bmjopen-2021-052628
Article Type:	Original research
Date Submitted by the Author:	21-Apr-2021

Complete List of Authors:	Dale, Maria Teresa; Norwegian Center for Violence and Traumatic Stress Studies; University of Oslo, Department of Psychology<br>Nissen, Alexander ; Norwegian Center for Violence and Traumatic Stress Studies<br>Berthelsen, Mona; Norwegian Center for Violence and Traumatic Stress Studies<br>Gjessing, Håkon ; Norwegian Institute of Public Health, Centre for Fertility and Health; University of Bergen, Department of Global Public Health and Primary Care, Faculty of Medicine<br>Heir, Trond; Norwegian Center for Violence and Traumatic Stress Studies; University of Oslo, Institute of Clinical Medicine, Faculty of Medicine

Keywords:	ACCIDENT & EMERGENCY MEDICINE, Health & safety < HEALTH SERVICES ADMINISTRATION & MANAGEMENT, MENTAL HEALTH, Adverse events < THERAPEUTICS

SCHOLARONE™
Manuscripts

I, the Submitting Author has the right to grant and does grant on behalf of all authors of the Work (as defined in the below author licence), an exclusive licence and/or a non-exclusive licence for contributions from authors who are: i) UK Crown employees; ii) where BMJ has agreed a CC-BY licence shall apply, and/or iii) in accordance with the terms applicable for US Federal Government officers or employees acting as part of their official duties; on a worldwide, perpetual, irrevocable, royalty-free basis to BMJ Publishing Group Ltd ("BMJ") its licensees and where the relevant Journal is co-owned by BMJ to the co-owners of the Journal, to publish the Work in this journal and any other BMJ products and to exploit all rights, as set out in our licence.

The Submitting Author accepts and understands that any supply made under these terms is made by BMJ to the Submitting Author unless you are acting as an employee on behalf of your employer or a postgraduate student of an affiliated institution which is paying any applicable article publishing charge ("APC") for Open Access articles. Where the Submitting Author wishes to make the Work available on an Open Access basis (and intends to pay the relevant APC), the terms of reuse of such Open Access shall be governed by a Creative Commons licence – details of these licences and which Creative Commons licence will apply to this Work are set out in our licence referred to above.

Other than as permitted in any relevant BMJ Author's Self Archiving Policies, I confirm this Work has not been accepted for publication elsewhere, is not being considered for publication elsewhere and does not duplicate material already published. I confirm all authors consent to publication of this Work and authorise the granting of this licence.

For peer review only

Title page

Psychosocial work factors and sick leave risk after a terrorist bomb attack: A prospective study among governmental employees in Norway.

Maria T. G. Dale[1,2*], Alexander F. Nissen[1], Mona Berthelsen[1], Håkon K. Gjessing [3,4], Trond Heir[1, 5]

[1] Norwegian Centre for Violence and Traumatic Stress Studies, Norway

[2] Department of Psychology, University of Oslo, Norway

[3] Centre for Fertility and Health, Norwegian Institute of Public Health

[4] Department of Global Public Health and Primary Care, Faculty of Medicine, University of Bergen

[5] Institute of Clinical Medicine, Faculty of Medicine, University of Oslo, Norway.

*Corresponding author: Norwegian Centre for Violence and Traumatic Stress Studies, Post box 181 Nydalen, 0409 Oslo, Norway. Phone: + 47 92 63 06 34. E- mail: m.t.g.dale@nkvts.no

Words 3.690

Keywords registry data; sick leave; work place terror; psychosocial work environment; social support

For peer review only

ABSTRACT

Objectives

Studies show that social support may reduce the negative psychological effects of terror. The aim was to explore prospectively the effects of the psychosocial work environment on sick leave risk among governmental employees after a workplace bomb attack.

Design

We linked survey data collected at 10 and 22 months after the bombing with doctor-certified sick leave data collected 42 months before the attack to 33 months after the attack. Odds ratios (ORs) and rate ratios (RRs) were estimated with mixed effects hurdle models.

Setting

The bombing of the government ministries in Oslo, Norway, 22 July 2011.

Participants

We identified 1625 participants from a cohort of 3520 employees working in the ministries during the bombing in 2011.

Results

After adjustment for confounders, social support from coworkers reduced the odds of sick leave (OR= 0.80, 95% CI 0.68-0.93), and there was marginal evidence for reduced odds with support from superior (OR= 0.87, 95% CI 0.87-1.03). A social work climate, an innovative climate and a human resource primacy climate (HRP) reduced the sick leave risk, with strongest estimates for HRP (OR= 0.77, 95% CI 0.66-0.90). The hurdle model found no associations between psychosocial support at work and the duration of sick leave.

Conclusions

For peer review only - http://bmjopen.bmj.com/site/about/guidelines.xhtml

Psychosocial support at work can enhance employees' work ability after terror and reduce the sick leave risk with more than 20 percent. However, a supportive psychosocial work environment did not reduce the duration of sickness absence. The protective role of psychosocial work factors on sick leave may be most significant when employees are at work and interact with their work environment.

Strengths and limitations of this study

- This study uses prospective objective registry data on doctor-certified sick leave in a large sample exposed to a workplace bomb attack.
- This study has a relatively high response rate and an appropriate number of participants with and without sick leave, making it possible to compare the two samples.
- In terms of generalizability, the study sample consisted of a majority of highly educated government officials and bureaucrats.
- Use of self-reported data on psychosocial work environment may not objectively reflect available social support.
- A comprehensive approach that incorporates both intrinsic and extrinsic factors is needed to better understand how individuals cope in the aftermath of terror.

For peer review only

INTRODUCTION

When terrorism strikes the workplace, where people spend much of their time, survivors are highly affected.[1] The workplace serves as an important social context that provides routines, purpose, economic and social resources to one's life, all of which can be impaired after workplace violence.[2] After the Oslo bombing in 2011, terror-exposed individuals were at high risk of posttraumatic stress disorder (PTSD), depression and increased sick leave.[3 4] However, many employees had high work

For peer review only - http://bmjopen.bmj.com/site/about/guidelines.xhtml

ability with a strong attachment and commitment to the workplace, and some managed to work despite high symptom load.[5] The magnitude of terror-related stressors might have different impact on survivors, where factors shown to affect the sickness absence are health, age, gender, coping style, personality, physical and psychosocial work factors, work schedule characteristics and available social resources. [6-8] In particular, it seems like the social network at work combined with a supportive organizational climate can be a significant source of support for employees.

Social support and good social relations affect health, and act as protective resources against the negative impact of major life events.[9 10] Social support involves instrumental (e.g., material and financial), informational (e.g., advice and guidance) and emotional (e.g., empathy, trust and emotional venting) resources.[11] Previous studies show that these three supportive components can be directly associated with overall lower levels of psychological distress, independent of exposure to trauma.[12] Additionally, social support can act as a buffer, by reducing or taking away the impact of a major trauma.[13] In line with this, studies show that after a natural disaster, low levels of social support have been associated to higher levels of PTSD and depression.[14-16] Further, when experiencing intense stress from terror, such as the September 11 terrorist attacks, research indicates that turning to others for help may prevent development of long-lasting psychological sequelae.[17] Although the social network at work is considered to be more formal than social relations to family and friends, research shows a strong association between support from colleagues and superiors and reduced sick leave.[18-20] However, the vast majority of studies on the effect of the psychosocial work environment on sick leave risk have not focused on workers exposed to a terror attack.

At present, it is unclear how the psychosocial work environment influences the sick leave risk and the duration of sick leave after terror. We assume that a high degree of social support from leaders and colleagues, combined with a supportive organizational climate with a concern for human

For peer review only

resources, are essential for employees struggling with returning to work after unexpected and threatening workplace violence. Our study sample shared an extraordinary experience and psychosocial support at work may be even more critical for reestablishing routines and recovery of trust and safety. The present study aims to explore whether increasing levels of psychosocial support are associated with a corresponding decrease in employees sick leave risk and reduction in sick leave days, and consequently add knowledge to the limited literature on this association.

METHODS

Study population and data sources

This prospective cohort study includes web-based survey-data combined with registry data on doctor certified sick leave from ministerial employees in 14 of 17 Ministries, after a car bomb attack at the Norwegian government offices in Oslo, July 22nd, 2011. The terror bombing caused substantial damage to buildings and infrastructure, killing eight and injuring 209 people. Negative health reactions were prevalent among all employees, and the present study includes all, whether indirectly or directly exposed to the bomb. [4]

The Norwegian Centre for Violence and Traumatic Stress Studies conducted the survey in collaboration with the National Institute of Occupational Health in Norway 10, 22 and 34 months after the terrorist attack. Eligible participants were informed about the study through their ministries, and received an invitation letter containing an unique log-in code to access the Web-based survey, including information on withdrawal procedures. Data on doctor certified sick leave was obtained from Statistics Norway and the Norwegian Labor and Welfare Administration.

For the purpose of this study, we used survey data on employees' background variables and information on psychosocial work environment 10 months after the attack (T1) and 22 months after

For peer review only - http://bmjopen.bmj.com/site/about/guidelines.xhtml

the attack (T2). At 34 months after the attack (T3) there was a major governmental change and reorganization following the 2013 Norwegian parliamentary election. This may have affected the subjective ratings on the psychosocial work environment, with change in leadership and organization culture, and therefore we did not include this period (T3) in the analysis. The survey data was linked with registry data on doctor certified sick leave from a period of 42 months before the attack to 33 months after the attack. Se Figure 1 *Timeline for sick leave outcomes and survey measures on psychosocial work environment* for an overview of the time line.

-Figure 1 about here-

All employees provided written consent, and strict procedures were followed to ensure confidentiality. 3520 invited employees consented to participate; 59 could not be reached with information about the study and 482 employees left the ministerial job or changed ministry affiliation prior to study completion. In this study population, we identified 1625 participants eligible for the study, all with relevant data on psychosocial work environment at T1 and T2 and data for the 9- months following T1 and T2. Based on the personal identification number from the Norwegian Population register, Statistics Norway performed data linkage and de-identification. Further details on design and participants has previously been described in a recent article from our research group.[3] See Figure 2 *Flow-chart displaying participant disposition* for more information on participants eligible for this study.

-Figure 2 about here-

Patient and public involvement

There is no direct patient involvement in this study. Neither patients nor public were directly involved the design, conduct, reporting or dissemination plans of our research.

For peer review only - http://bmjopen.bmj.com/site/about/guidelines.xhtml

Psychosocial work exposures

Social support at work (comprising two subscales) and a supportive organizational culture (comprising three subscales), were measured by the General Nordic Questionnaire for Psychological and Social Factors at Work, QPS_{Nordic}. [21] All responses were scored on an ordinal five-point scale ranging from "1 = very seldom or never" to "5 = very often or always", and missing response to one of the items comprising each scale was allowed.

Social support from coworkers was measured with 2 items, and a typical item was "If needed, can you get support and help with your work from your coworkers?" Social support from superior comprised 3 items. A typical item was "If needed, is your immediate superior willing to listen to your work-related problems?" Three subscales measured organizational culture: social climate, human resource primacy climate (HRP) and innovative climate, all comprising 3 items. An item representing social organizational climate was "Is the climate encouraging and supportive in your work unit?" A typical item measuring HRP was "Are workers well taken care of in your organization?" To measure innovative climate a relevant item was "Are workers encouraged to think of ways to do things better at your workplace?" Reliability tests of the five subscales have demonstrated approvable internal consistency measured with Chronbach's alpha from .71 to .83 and test-retest reliability from .72 to .83 with more than five weeks interval.[22]

Sick leave

The outcome of this study, doctor-certified sickness absence, was based on registry data on employment from Statistics Norway and registry data on sick-leave from the Norwegian Labour and Welfare Administration. The former registry contains the number of expected workdays per quarter for a given person based on the person's contract(s) of employment. Weekends, public holidays and

For peer review only - http://bmjopen.bmj.com/site/about/guidelines.xhtml

days of vacation are not considered potential workdays. For a person with full-time employment, there are roughly 170 expected workdays in each 9-month period explored in the study. The registry on sick-leave contains the number of days a given person was absent from work per quarter due to doctor-certified sick-leave. The registry takes account of whether the person works full-time or part-time, and whether sick-leave was graded or not (e.g. a person may be on 50% sick-leave). For example, if a person with 80% employment (i.e. four expected workdays per week) gets one week of 50%, doctor-certified sick-leave in a quarter, the person will have 2 registered days of sickness absence for that quarter. Sick leave prior to the attack (from first quarter of 2008 up until the second quarter of 2011) was included as a potential confounder, and defined as total sickness absence days divided by the number of expected work-days registered for this period. When used as an outcome variable in the main analysis, sick leave was examined in the two nine-month periods following the survey points at T1 and T2 (see Figure 1 for time frames).

Covariates

Covariates that could potentially influence the association between the psychosocial work environment and sick leave risk were considered a priori as potential confounders. The following covariates were included in the main analysis: time, sex, age, education, direct versus indirect exposure to terror and sick leave prior to the attack.

Analytic strategy

Mixed effects hurdle models were used in analyses, and participants contributed with data as long as they had at least one time point without missing values.

For peer review only - http://bmjopen.bmj.com/site/about/guidelines.xhtml

Frequency of sick leave was expressed as count data, with excess of zeros (no sick leave).[23] As the count data was over-dispersed with variance larger than the mean, we used negative binomial hurdle models to estimate the incidence rate of sick leave.[24 25] Hurdle models are two-part models. The first part uses a logistic regression model to estimate the odds of the outcome being above zero vs. zero for various predictor levels in the model, summarized as odds ratios (ORs) compared to a set reference. In the present study, we estimated the odds ratio of sick leave among employees according to various psychosocial work environments, using crude (OR) and adjusted (aOR) odds ratios with 95% confidence intervals. The ORs compare the odds of having at least one day of sickness absence vs. no sickness absence for various predictor levels. The second part of the hurdle model studies the mean number of days of sickness absence, conditional on having at least one day of absence. For this part, it uses a truncated negative binomial regression model, where zero has been excluded, for the positive counts.[26] By fitting the negative binomial model we obtain mean count ratios, or rate ratios (RR, with 95% CIs). The rate ratio compares the mean number of days of sickness absence over levels of various predictors, including the main exposure. Consequently, the RRs estimate the exposure effect on the number of sickness absence days among those who have at least one day of absence.

To account for varying person-time at risk (i.e. not all employees worked full-time during the observation period), the hurdle models were offset by employees' expected workdays in the relevant periods. Since data were collected longitudinally, with repeated measurements on individuals, we used a mixed effects extension of the hurdle model, with a random intercept on individuals.

The binomial regression analyses and the zero-truncated negative binominal analysis were computed in STATA version 15 (STATA Corporation, College Station, TX, USA) and R, using the R package GLMMadaptive.

For peer review only - http://bmjopen.bmj.com/site/about/guidelines.xhtml

RESULTS

Characteristics of the Study Population

In the study population of 1,625 employees, the prevalence of sick leave from 13 to 21 months after the attack was 259/1000 (421/1,625) after exclusion of employees leaving their job or changing ministry affiliation prior to study completion. After the second wave of the survey (T2) in the period from 25 months to 33 months after the attack the sick leave prevalence was 242/1000 (394/1,625). See Figure 1. Further, the mean number of sickness absence days was about 25 days at 13 to 24 months after the first survey (T1) and decreased to 22 days in the period of 25 to 36 months after the second wave of the survey (T2) among employees with sick leave. When expressed as overall percentage among all employees in this study, this corresponds to 4.3 and 3.8 percent sick leave of all working days.

Table 1 compares participants with and without sick leave 10 months after the attack (T1) and 22 months after the attack (T2). At both measurement points (T1 and T2) the group with sick leave had overall lower levels of education ($p < .001$), higher scores in psychological distress ($p < .001$) and higher symptom scores for post-traumatic stress ($p < .001$), and higher percentage of females (68%) compared to the group without sick leave. At T2 the participants were 1.3 years older among the group with sick leave ($p= .043$) (than those without sick leave at same time point,) and at T1 there were more participants directly exposed to the bomb explosion among those with sick leave (13%) than those without sick leave (9%). The groups with sick leave did not differ significantly from participants without sick leave with regard to marital status at T1 and T2. Table 1 *Characteristics in cases with no sick leave and those with sick leave* shows the distribution of background variables for sex, age, educational level, relationship status, directly- and indirectly exposed, psychological distress and sick leave, all retrieved from the questionnaire survey completed 10 months (T1) and 22 months (T2) after the terror attack.

For peer review only - http://bmjopen.bmj.com/site/about/guidelines.xhtml

-Table 1 about here-

Psychosocial support at work and sick leave risk

After adjusting for confounders we observed that high support from coworkers was associated with overall reduced odds of sick leave for all time periods (aOR = 0.80, 95% CI 0.68-0.93). Similarly, support from superior indicates reduced odds of sick leave (aOR = 0.87, 95% CI 0.79-1.01), but did not reach significance in the model (p=.063). A work environment with a social, innovative and human resource primacy climate reduced the odds of sick leave, with strongest estimates for HRP (OR=0.77, 95% CI 0.66-0.90). Moreover, the hurdle model estimated the duration of sick leave (RR) among those with sick leave. We found no significant associations between social support from superior and coworkers and the duration of sickness absence among those with sick leave, nor between a supportive HRP, social climate and an innovative organizational culture and the duration of sickness absence among those with sick leave (see Table 2 *Two-part hurdle mixed effects models on work factors*). The RR-estimates indicate that all five factors measuring a supportive psychosocial work environment had no substantial impact on the duration of sick leave. Further, the overall effects of Time (T1 to T2) on sick leave scores after the attack were not significant (e.g. OR = 0.94, 95% CI 0.76-1.18 and RR = 0.85, 95% CI 0.71-1.02) indicating unchanged odds and rate ratio for sick leave at T2 when compared to T1 (the estimates for confounders are not shown in Table 2).

-Table 2 about here-

DISCUSSION

This is the first cohort study based on register data on sick leave exploring longitudinally whether a supportive psychosocial work environment reduces the risk and duration of sickness absence after a

For peer review only - http://bmjopen.bmj.com/site/about/guidelines.xhtml

terrorist attack on the workplace. Our findings indicate that a supportive psychosocial work environment can reduce the odds for sick leave with more than 20 percent. For the second part of the Hurdle-analysis, we observed no associations between a supportive psychosocial work environment and the duration of sickness absence. Also of relevance, we found that employees with sick leave had significantly more psychological strains and symptoms compared to those without sick leave, and they were older with proportionally more women.

Our first finding is in accordance with other studies (without focus on terrorism exposure) that find a protective effect of social support from the work environment, leading to reduced risk of sick leave.[6-8] Especially, colleague support and appropriate supervision from the leader are important.[27 28] Moreover, previous findings show that high levels of social support at work are associated with reduced psychological distress, depression and increased well-being.[10 12-14] Evidence for the protective effect of social support on mental health and distress is relevant for our outcome on sick leave risk, as employment and sick leave frequency are markers of functional recovery from a trauma. Most people affected by disasters do not develop severe psychiatric disorders, though almost everyone with exposure to disaster trauma will experience distress for at least a brief period. A measure on sickness absence can capture individuals with sub-diagnostic distress after terrorism.

Our second main finding showed that psychosocial support was not associated with a reduction in the duration of sickness absence, indicating that the protective role of a supportive psychosocial work environment was significant only when employees managed to stay at work. This can partly be explained by studies showing that the relationship between social support at work and sickness absence is bidirectional or reciprocal.[18 29] A study by Sieurin et al (2009) found that long-term absentees often reported that their absence negatively affected their sense of belonging to the social workgroup.[30] One speculation is that sickness absence may affect the social relationships at work, and

For peer review only - http://bmjopen.bmj.com/site/about/guidelines.xhtml

thereby adding to the challenges causing the sickness absence in the first place. Another factor could simply be that employees absent from work lose essential interaction with their work environment, and consequently the positive effects of psychosocial support are reduced. Further, there might have been a "threshold effect" in our sample, where employees with sick leave had reached a higher level of adverse health and distress, where coping strategies and support from the work environment no longer have effect on frequency of sickness absence days.

The utility of social resources depends on the survivors' ability to seek and receive support from their social and interpersonal resources in the aftermath of a terror attack.[14] Clearly, employees with sick leave had significantly more psychological strains and symptoms compared to those without sick leave, and they were older with proportionally more women, which is in line with previous findings on sick leave risk with evidence for gender, older age and previous history of sickness as risk factors.[17 31-33] According to the social selection hypothesis, people struggling with post-traumatic stress symptoms such as withdrawal, depression and irritability may not seek necessary social support but instead diminish their interpersonal relations over time.[12 34] Further, employees experiencing psychological distress might evaluate the psychosocial work environment more negatively. This explanation can be related to a tendency in our results where leader support was not the most essential protective factor against sick leave for the employees after the terror attack. It could be that employees suffering from psychological distress after the attack perceived their immediate leader to be less supportive, as distress could have a negative effect on subjective appraisals of leader support. [35]

Strengths and limitations

By means of a unique longitudinal design with registry data on sick leave, we were able to extend our knowledge of sick leave risk, and how it relates to the psychosocial work environment after terror.

For peer review only - http://bmjopen.bmj.com/site/about/guidelines.xhtml

Existing research on terrorism and health effects are mostly focused on psychotraumatology and mental disorders such as PTSD, and sick leave may be a better objective measure of general functioning and health, especially consistently and routinely collected doctor certified data. [7 36]

The study has several limitations. First, the study population consisted of a majority of highly educated government officials and bureaucrats and our findings may not necessarily be generalized to other populations. It could be that the terror attack could have greater negative health effects in another sample, as the study sample was highly educated with flexible jobs, which is associated with lower incidence rate of sick leave.[37] Second, we used data on doctor-certified sick leave. In Norway the workers are entitled to be home from work up to three consecutive days, four times per year, without doctor certification. Therefore, the overall incidence rate of sick leave would be higher if this was included in the official registry. Third, the assessment of the psychosocial work environment was based on self-reported measures and may be influenced by social desirability, underreporting, recall bias and other response bias, where subjective appraisals of the work environment are closely linked to personality traits and negative affect.[38] However, the QPS_{Nordic} has been validated as an effective instrument for measuring psychological and social factors at work and should be relatively insensitive to personality and emotions, for example: respondents were asked about how frequent a situation occurs instead of degrees of agreement or satisfaction.[22] Finally, we had no information on workplace social support prior to the terrorist attack, allowing comparison of the effect of social support on sick leave before and after the terrorist attack. However, we had data on sick leave before the attack and were able to adjust for important confounders, such as previous sickness absence in the main analysis, a factor that could potentially influence later sick leave risk and participant ratings of the psychosocial work environment.

For peer review only - http://bmjopen.bmj.com/site/about/guidelines.xhtml

Implications

It seems reasonable to conclude that psychosocial support at work can reduce the sick leave risk after terror. The workplace should mobilize its social and supportive resources before employees become sick listed, and before potentially new negative psychological and physical reactions have time to develop and at worse result in long-term sick leave. Especially as the psychosocial work factors appeared to have no major impact on the duration of sickness absence. Future research should investigate whether the associations are causal, and whether these findings can be replicated in other populations. A more comprehensive approach that incorporates both intrinsic and extrinsic factors is needed to better capture and understand how individuals cope in the aftermath of terror and to identify the difficulties for return to work.

Acknowledgements We thank all the governmental employees who took part in this study.

Author contributions TH designed the study. MTGD and AFN analyzed the data and MTGD drafted the paper. All authors, TH, MB, HG, AFN and MTGD, participated in project meetings where the analysis plan and data interpretation were discussed, and where the article was critically revised.

Funding This work was funded by grants from Research Council Norway, grant number 227039.

Conflict of interests None declared.

Patient consent for publication Not required.

For peer review only - http://bmjopen.bmj.com/site/about/guidelines.xhtml

Ethical approval Informed consent was obtained from all participants, and the Regional Committee for Ethics in Medical Research approved the study (reference number: 2011/1577).

Provenance and peer review Not commissioned; externally peer reviewed.

Data sharing statement Data are available from the corresponding author on reasonable request. The datasets generated during and/or analyzed during the current study are not publicly available due to confidentiality agreements made with participants.

ORCID iD Maria Teresa Grønning Dale https://orcid.org/0000-0002-0972-2996

REFERENCES

1. North CS, Pfefferbaum B, Hong BA, et al. The Business of Healing: Focus Group Discussions of Readjustment to the Post-9/11 Work Environment Among Employees of Affected Agencies. *Journal of Occupational and Environmental Medicine* 2010;52(7):713-18. doi: 10.1097/JOM.0b013e3181e48b01
2. Weiseth L, Heir T. Workplace and organizational disasters: response and planning. In: Ursano R, (Ed.), Textbook of disaster psychiatry. Cambridge: Cambridge University Press 2017:261-69.
3. Hansen MB, Berthelsen M, Nissen A, et al. Sick leave before and after a work-place targeted terror attack. *International archives of occupational and environmental health* 2019;92(3):327-35. doi: 10.1007/s00420-018-1390-8
4. Hansen MB, Birkeland MS, Nissen A, et al. Prevalence and Course of Symptom-Defined PTSD in Individuals Directly or Indirectly Exposed to Terror: A Longitudinal Study. *Psychiatry* 2017;80(2):171-83. doi: 10.1080/00332747.2016.1230983
5. Dale MTG, Nissen A, Berthelsen M, et al. Post-traumatic stress reactions and doctor-certified sick leave after a workplace terrorist attack: a Norwegian cohort study. *BMJ open* 2020; 10:e032693. doi: 10.1136/bmjopen-2019-032693
6. Boot CRL, van Drongelen A, Wolbers I, et al. Prediction of long-term and frequent sickness absence using company data. *Occupational Medicine* 2017;67(3):176-81. doi: 10.1093/occmed/kqx014
7. Melchior M, Niedhammer I, Berkman LF, et al. Do psychosocial work factors and social relations exert independent effects on sickness absence? A six year prospective study of the GAZEL cohort. *Journal of Epidemiology and Community Health* 2003;57(4):285-93. doi: 10.1136/jech.57.4.285
8. Michie S, Williams S. Reducing work related psychological ill health and sickness absence: a systematic literature review. *Occup Environ Med* 2003;60(1):3-9. doi: 10.1136/oem.60.1.3

For peer review only - http://bmjopen.bmj.com/site/about/guidelines.xhtml

9. Diener E, Seligman MEP. Very Happy People. *Psychological Science* 2002;13(1):81-84. doi: 10.1111/1467-9280.00415
10. Taylor SE. Social support: A review. The Oxford handbook of health psychology. In H. S. Friedman (Ed.), The Oxford handbook of health psychology. New York, NY: Oxford University Press 2011:189-214.
11. Cohen S. Social Relationships and Health. Am Psychol 2004; 59: 676–684. https://doi.org/10.1037/0003-066X.59.8.676
12. Santini ZI, Koyanagi A, Tyrovolas S, et al. The association between social relationships and depression: A systematic review. *Journal of Affective Disorders* 2015;175:53-65. doi: https://doi.org/10.1016/j.jad.2014.12.049
13. Cohen S, Wills TA. Stress, social support, and the buffering hypothesis. *Psychol Bull* 1985;98(2):310-57. [published Online First: 1985/09/01]
14. Arnberg FK, Hultman CM, Michel P-O, et al. Social Support Moderates Posttraumatic Stress and General Distress After Disaster. *Journal of Traumatic Stress* 2012;25(6):721-27. doi: 10.1002/jts.21758
15. Fullerton CS, Ursano RJ, Kao T-C, et al. Diaster-realted bereavement: Acute symptoms and subsequent depression. *Aviation, Space, and Environmental Medicine* 1999;70(9):902-09.
16. Madakasira S, O'Brien KF. Acute posttraumatic stress disorder in victims of a natural disaster. *Journal of Nervous and Mental Disease* 1987;175(5):286-90. doi: 10.1097/00005053-198705000-00008
17. Galea S, Ahern J, Resnick H, et al. Psychological Sequelae of the September 11 Terrorist Attacks in New York City. *New England Journal of Medicine* 2002;346(13):982-87. doi: 10.1056/NEJMsa013404
18. Knapstad M, Holmgren K, Hensing G, et al. Previous sickness absence and current low perceived social support at work among employees in the general population: a historical cohort study. *BMJ open* 2014;4(10):e005963. doi: 10.1136/bmjopen-2014-005963
19. Vahtera J, Kivimäki M, Pentti J, et al. Effect of change in the psychosocial work environment on sickness absence: a seven year follow up of initially healthy employees. *Journal of epidemiology and community health* 2000;54(7):484-93. doi: 10.1136/jech.54.7.484
20. Moreau M, Valente F, Mak R, et al. Occupational stress and incidence of sick leave in the Belgian workforce: the Belstress study. *Journal of Epidemiology and Community Health* 2004;58(6):507-16. doi: 10.1136/jech.2003.007518
21. Dallner M EA-L, Gamberale F, Hottinen V, Knahrdahl S, Lindtröm K , et al Validation of the General Nordic Questionnaire (QPSNordic) for psychological and social factors at work. *Copenhagen: Nordic Council of Ministers, 2000*;NORD 2000:12
22. Lindström K, Ørhede E, Elo A-L, et al. User's guide for the QPSNordic : General Nordic Questionnaire for Psychological and Social Factors at Work. København: Nordic Council of Ministers 2000.
23. Cameron AC, Trivedi PK. Regression analysis of count data. Second ed. Cambridge, UK: Cambridge University Press 2013.
24. Cheung YB. Zero-inflated models for regression analysis of count data: a study of growth and development. *Statistics in Medicine* 2002;21(10):1461-69. doi: 10.1002/sim.1088
25. Zeileis A. KC, Jackman S. Regression Models for Count Data in R. *Journal of Statistical Software* 2008;27(8) doi: Available at: http://dx.doi.org/10.18637/jss.v027.i08.
26. Rodriguez G. Models for count data with overdispersion. Retrieved from Princeton University: 2013, November 6th. *http://dataprincetonedu/wws509/notes/c4apdf*

For peer review only - http://bmjopen.bmj.com/site/about/guidelines.xhtml

27. Finne LB, Christensen JO, Knardahl S. Psychological and social work factors as predictors of mental distress: a prospective study. *PloS one* 2014;9(7):e102514-e14. doi: 10.1371/journal.pone.0102514
28. Kuoppala J, Lamminpää A, Liira J, et al. Leadership, Job Well-Being, and Health Effects—A Systematic Review and a Meta-Analysis. *Journal of Occupational and Environmental Medicine* 2008;50(8):904-15. doi: 10.1097/JOM.0b013e31817e918d
29. Hausser JA, Mojzisch A, Niesel M, et al. Ten years on: A review of recent research on the Job Demand-Control (-Support) model and psychological well-being. *Work & Stress* 2010;24(1):1-35. doi: 10.1080/02678371003683747
30. Sieurin L, Josephson M, Vingård E. Positive and negative consequences of sick leave for the individual, with special focus on part-time sick leave. *Scandinavian Journal of Public Health* 2008;37(1):50-56. doi: 10.1177/1403494808097171
31. Dekkers-Sánchez PM, Hoving JL, Sluiter JK, et al. Factors associated with long-term sick leave in sick-listed employees: a systematic review. *Occup Environ Med* 2008;65(3):153-57. doi: 10.1136/oem.2007.034983
32. Mastekaasa A. The gender gap in sickness absence: long-term trends in eight European countries. *European Journal of Public Health* 2014;24(4):656-62. doi: 10.1093/eurpub/cku075
33. North CS, Nixon SJ, Shariat S, et al. Psychiatric Disorders Among Survivors of the Oklahoma City Bombing. *JAMA* 1999;282(8):755-62. doi: 10.1001/jama.282.8.755
34. Steenstra IA, Verbeek JH, Heymans MW, et al. Prognostic factors for duration of sick leave in patients sick listed with acute low back pain: a systematic review of the literature. *Occup Environ Med* 2005;62(12):851-60. doi: 10.1136/oem.2004.015842
35. Birkeland MS, Nielsen MB, Hansen MB, et al. The impact of a workplace terrorist attack on employees' perceptions of leadership: A longitudinal study from pre- to postdisaster. *The Leadership Quarterly* 2017;28(5):659-71. doi: https://doi.org/10.1016/j.leaqua.2017.01.002
36. Kivimäki M, Head J, Ferrie JE, et al. Sickness absence as a global measure of health: evidence from mortality in the Whitehall II prospective cohort study. *BMJ* 2003;327(7411):364. doi: 10.1136/bmj.327.7411.364
37. Robroek SJW, van Lenthe FJ, Burdorf A. The role of lifestyle, health, and work in educational inequalities in sick leave and productivity loss at work. *International archives of occupational and environmental health* 2013;86(6):619-27. doi: 10.1007/s00420-012-0793-1
38. Wainwright D, Calnan M. Work stress: the making of a modern epidemic. Buckingham: Open University Press, 2002:21–3.

For peer review only

For peer review only - http://bmjopen.bmj.com/site/about/guidelines.xhtml

Table 1 Characteristics in cases with no sick leave and those with sick leave at 10 months (T1) and 22 months (T2) after the 2011 terrorist attack in the cohort with 1,625 Norwegian ministerial employees.

	T1				T2			
**Characteristics**	**No sick leave n = 1023**	**Sick leave n = 349**	**χ^2/F**	**p[a]**	**No sick leave n= 990**	**Sick leave n= 324**	**χ^2/F**	**p[a]**
Females, n (%)	528 (51.6)	248 (71.1)*	40.1	<0.001	525 (53.0)	229 (70.7)*	31.1	<0.001
Education, n (%)			41.7	<0.001			38.4	<0.001
< 13 years	82 (8.0)	68 (19.5)*			92 (9.4)	72 (22.2)*		
13-16 years	224 (21.9)	89 (25.5)*			218 (22.1)	73 (22.5)		
> 16 years	717 (70.1)	192 (55.0)*			677 (68.5)	179 (55.3)*		
Married/Cohabiting, n (%)	598 (75.5)	183 (68.3)	5.4	0.02	730 (73.7)	245 (75.9)	0.6	0.45
Age (M ± SD)	46.3 ± 10.6	47.3 ± 10.5	2.3	0.14	46.4 ± 10.4	47.6 ± 10.7	3.2	0.07
Directly exposed, n (%)	100 (9.8)	49 (14.0)*	4.9	0.03	112 (11.4)	32 (9.9)	0.5	0.47
Psychological distress, score 1-4 (M ± SD)	1.24 ± 0.39	1.54 ± 0.61*	110.5	<0.001	1.27 ± 0.42	1.48 ± 0.6*	50.0	<0.001
PCL-based PTSD, score 1-5 (M ± SD)	1.34 ± 0.46	1.72 ± 0.76*	118.2	<0.001	1.28 ± 0.46	1.51 ± 0.68*	44.6	<0.001
Sick-leave days (M ± SD) [b]		24.8 ± 34.6				20.8 ± 25.7		

Table showing case numbers and within-group percentages
*Differs significantly from those without sick leave
[a] P values were calculated using ANOVA for continuous variables and χ^2 test for categorical variables.
[b] Nine-months period following the survey questionnaires at T1 and T2
M Mean; SD Standard Deviation

For peer review only

For peer review only - http://bmjopen.bmj.com/site/about/guidelines.xhtml

Table 2 Two-part hurdle mixed effects models on work factors among 1,625 Norwegian ministerial employees measured 10 and 22 months after the terrorist attack 22nd of July, 2011, and risk of sick leave measured nine-month after each survey.

	Part I: Binary logistic model		Part II: Negative binomial count model	
**Variables**	**OR**	**OR[a]**	**RR**	**RR[a]**
**Psychosocial work environment**				
Social support/interactions:				
Support from superior	0.81 (0.70, 0.93)[1]	0.87 (0.87, 1.03)	0.90 (0.80, 1.00)	0.94 (0.84, 1.05)
Support from coworkers	0.75 (0.64, 0.89)[2]	0.80 (0.68, 0.93)[1]	0.89 (0.79, 1.01)	0.94 (0.83, 1.07)
Organizational culture:				
Social organizational climate	0.72 (0.59, 0.86)[2]	0.78 (0.66, 0.93)[4]	0.98 (0.85, 1.12)	1.03 (0.90, 1.18)
Human resource primacy climate	0.70 (0.59, 0.83)[2]	0.77 (0.66, 0.90)[2]	0.88 (0.77, 1.00)	0.93 (0.82, 1.05)
Innovative organizational climate	0.79 (0.66, 0.95)[3]	0.82 (0.69, 0.98)[5]	0.95 (0.84, 1.09)	0.99 (0.86, 1.13)

OR Odds Ratio [95% Confidence Interval (CI)] for sickness absence from work (Yes/No)

RR Rate Ratio (95% CI) for number of sickness absence days among those with sick leave

[a] adjusted for time, sex, age, education, sick leave prior to attack and direct exposure to the bomb explosion.

[1] p = 0.005; [2] p < 0.001; [3] p = 0.014; [4] p = 0.006; [5] p = 0.02

For peer review only - http://bmjopen.bmj.com/site/about/guidelines.xhtml

Figure 1 Timeline for sick leave outcomes and survey measures on psychosocial work environment

- Uploaded separately in PDF format-

Figure 2 Flow-chart displaying participant disposition

- Uploaded separately in PDF format-

For peer review only

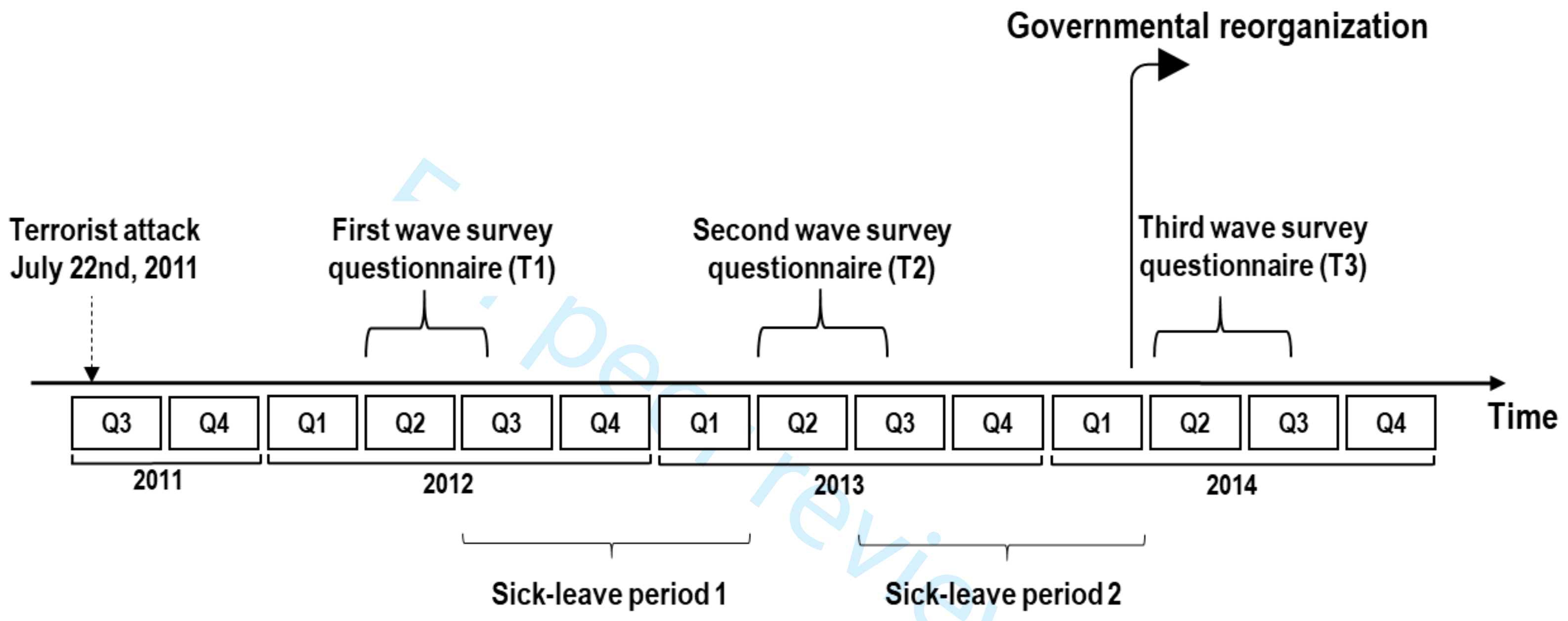

Q: Quarter
T1: Starts 10 months after the attack
T2: Starts 22 months after the attack
T3: Starts 34 months after the attack

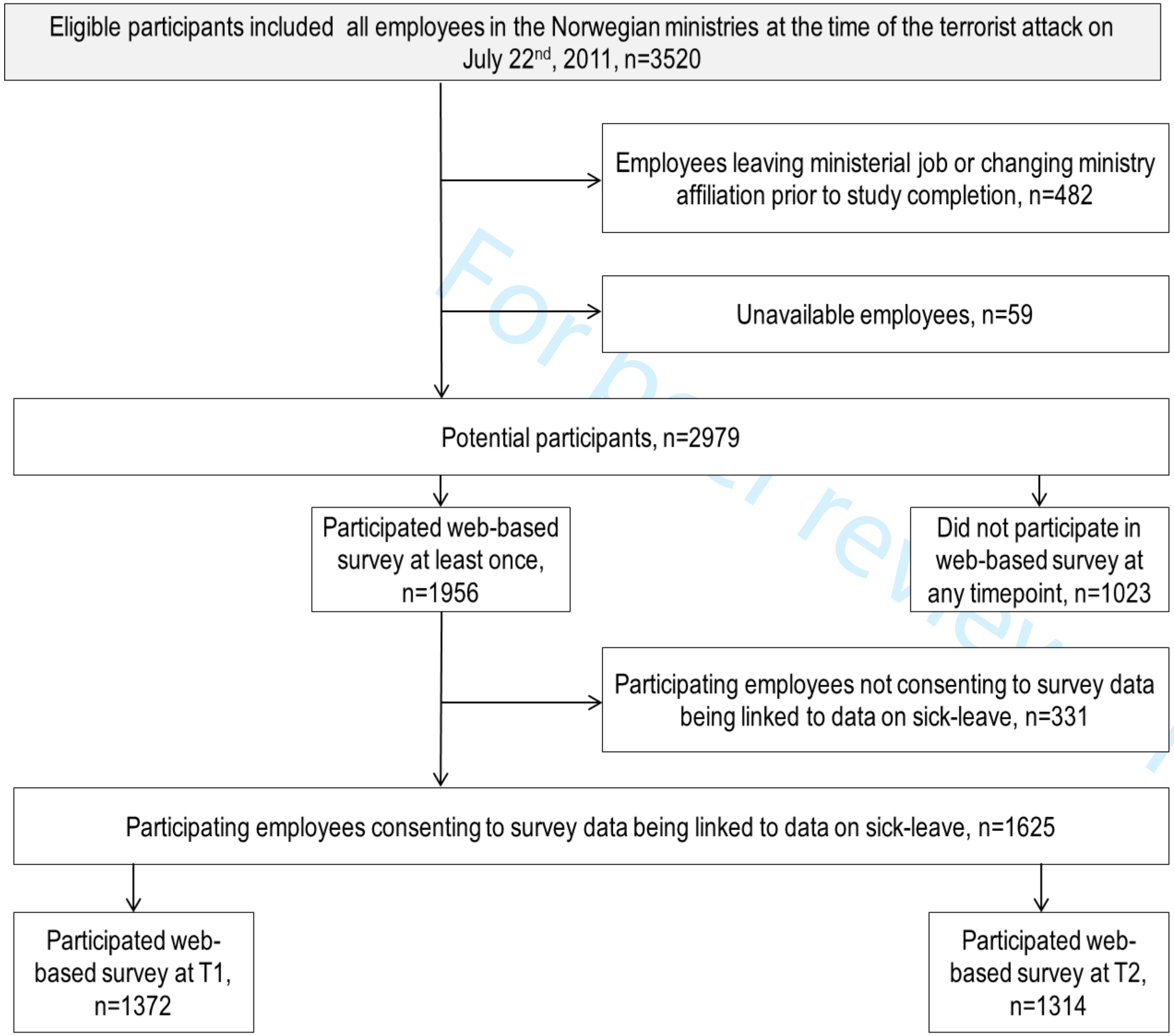
Eligible participants included all employees in the Norwegian ministries at the time of the terrorist attack on July 22nd, 2011, n=3520
Employees leaving ministerial job or changing ministry affiliation prior to study completion, n=482
Unavailable employees, n=59
Potential participants, n=2979
Participated web-based survey at least once, n=1956
Did not participate in web-based survey at any timepoint, n=1023
Participating employees not consenting to survey data being linked to data on sick-leave, n=331
Participating employees consenting to survey data being linked to data on sick-leave, n=1625
Participated web-based survey at T1, n=1372
Participated web-based survey at T2, n=1314

STROBE Statement—Checklist of items that should be included in reports of ***cohort studies***

	Item No	Recommendation	Page No
**Title and abstract**	1	(*a*) Indicate the study's design with a commonly used term in the title or the abstract	1
		(*b*) Provide in the abstract an informative and balanced summary of what was done and what was found	1-2
**Introduction**			
Background/rationale	2	Explain the scientific background and rationale for the investigation being reported	3-4
Objectives	3	State specific objectives, including any prespecified hypotheses	4
**Methods**			
Study design	4	Present key elements of study design early in the paper	4-5
Setting	5	Describe the setting, locations, and relevant dates, including periods of recruitment, exposure, follow-up, and data collection	4-5 Figure1
Participants	6	(*a*) Give the eligibility criteria, and the sources and methods of selection of participants. Describe methods of follow-up	4-5
		(*b*) For matched studies, give matching criteria and number of exposed and unexposed	n/a
Variables	7	Clearly define all outcomes, exposures, predictors, potential confounders, and effect modifiers. Give diagnostic criteria, if applicable	6-7
Data sources/ measurement	8*	For each variable of interest, give sources of data and details of methods of assessment (measurement). Describe comparability of assessment methods if there is more than one group	6-7
Bias	9	Describe any efforts to address potential sources of bias	7
Study size	10	Explain how the study size was arrived at	7-8 and Figure 2
Quantitative variables	11	Explain how quantitative variables were handled in the analyses. If applicable, describe which groupings were chosen and why	7-8
Statistical methods	12	(*a*) Describe all statistical methods, including those used to control for confounding	4-8
		(*b*) Describe any methods used to examine subgroups and interactions	n/a (not relevant)
		(*c*) Explain how missing data were addressed	9 Figure 2
		(*d*) If applicable, explain how loss to follow-up was addressed	9 Figure 2
		(*e*) Describe any sensitivity analyses	n/a
**Results**			
Participants	13*	(a) Report numbers of individuals at each stage of study—eg numbers potentially eligible, examined for eligibility, confirmed eligible, included in the study, completing follow-up, and analysed	9 Figure 2
		(b) Give reasons for non-participation at each stage	9
		(c) Consider use of a flow diagram	
Descriptive data	14*	(a) Give characteristics of study participants (eg demographic, clinical, social) and information on exposures and potential confounders	
		(b) Indicate number of participants with missing data for each variable of interest	
		(c) Summarise follow-up time (eg, average and total amount)	

For peer review only

Outcome data	15*	Report numbers of outcome events or summary measures over time	

For peer review only

For peer review only - http://bmjopen.bmj.com/site/about/guidelines.xhtml

Main results	16	(*a*) Give unadjusted estimates and, if applicable, confounder-adjusted estimates and their precision (eg, 95% confidence interval). Make clear which confounders were adjusted for and why they were included<br>(*b*) Report category boundaries when continuous variables were categorized<br>(*c*) If relevant, consider translating estimates of relative risk into absolute risk for a meaningful time period	
Other analyses	17	Report other analyses done—eg analyses of subgroups and interactions, and sensitivity analyses	
**Discussion**			
Key results	18	Summarise key results with reference to study objectives	
Limitations	19	Discuss limitations of the study, taking into account sources of potential bias or imprecision. Discuss both direction and magnitude of any potential bias	
Interpretation	20	Give a cautious overall interpretation of results considering objectives, limitations, multiplicity of analyses, results from similar studies, and other relevant evidence	
Generalisability	21	Discuss the generalisability (external validity) of the study results	
**Other information**			
Funding	22	Give the source of funding and the role of the funders for the present study and, if applicable, for the original study on which the present article is based	

*Give information separately for exposed and unexposed groups.

Note: An Explanation and Elaboration article discusses each checklist item and gives methodological background and published examples of transparent reporting. The STROBE checklist is best used in conjunction with this article (freely available on the Web sites of PLoS Medicine at http://www.plosmedicine.org/, Annals of Internal Medicine at http://www.annals.org/, and Epidemiology at http://www.epidem.com/). Information on the STROBE Initiative is available at http://www.strobe-statement.org.

For peer review only

BMJ Open

Psychosocial work factors and sick leave risk after a terrorist bomb attack: a survey and registry-based longitudinal study of governmental employees in Norway.

Journal:	*BMJ Open*
Manuscript ID	bmjopen-2021-052628.R1
Article Type:	Original research
Date Submitted by the Author:	06-Sep-2021
Complete List of Authors:	Dale, Maria Teresa; Norwegian Center for Violence and Traumatic Stress Studies; University of Oslo, Department of Psychology<br>Nissen, Alexander ; Norwegian Center for Violence and Traumatic Stress Studies<br>Berthelsen, Mona; Norwegian Center for Violence and Traumatic Stress Studies<br>Gjessing, Håkon ; Norwegian Institute of Public Health, Centre for Fertility and Health; University of Bergen, Department of Global Public Health and Primary Care, Faculty of Medicine<br>Heir, Trond; Norwegian Center for Violence and Traumatic Stress Studies; University of Oslo, Institute of Clinical Medicine, Faculty of Medicine
**Primary Subject Heading**:	Occupational and environmental medicine
Secondary Subject Heading:	Occupational and environmental medicine, Epidemiology, Mental health
Keywords:	ACCIDENT & EMERGENCY MEDICINE, Health & safety < HEALTH SERVICES ADMINISTRATION & MANAGEMENT, MENTAL HEALTH, Adverse events < THERAPEUTICS

SCHOLARONE™
Manuscripts

I, the Submitting Author has the right to grant and does grant on behalf of all authors of the Work (as defined in the below author licence), an exclusive licence and/or a non-exclusive licence for contributions from authors who are: i) UK Crown employees; ii) where BMJ has agreed a CC-BY licence shall apply, and/or iii) in accordance with the terms applicable for US Federal Government officers or employees acting as part of their official duties; on a worldwide, perpetual, irrevocable, royalty-free basis to BMJ Publishing Group Ltd ("BMJ") its licensees and where the relevant Journal is co-owned by BMJ to the co-owners of the Journal, to publish the Work in this journal and any other BMJ products and to exploit all rights, as set out in our licence.

The Submitting Author accepts and understands that any supply made under these terms is made by BMJ to the Submitting Author unless you are acting as an employee on behalf of your employer or a postgraduate student of an affiliated institution which is paying any applicable article publishing charge ("APC") for Open Access articles. Where the Submitting Author wishes to make the Work available on an Open Access basis (and intends to pay the relevant APC), the terms of reuse of such Open Access shall be governed by a Creative Commons licence – details of these licences and which Creative Commons licence will apply to this Work are set out in our licence referred to above.

Other than as permitted in any relevant BMJ Author's Self Archiving Policies, I confirm this Work has not been accepted for publication elsewhere, is not being considered for publication elsewhere and does not duplicate material already published. I confirm all authors consent to publication of this Work and authorise the granting of this licence.

For peer review only

Title page

Psychosocial work factors and sick leave risk after a terrorist bomb attack: a survey and registry-based longitudinal study of governmental employees in Norway.

Maria T. G. Dale[1,2*], Alexander F. Nissen[1], Mona Berthelsen[1], Håkon K. Gjessing[3,4], Trond Heir[1, 5]

[1] Norwegian Centre for Violence and Traumatic Stress Studies, Norway

[2] Department of Psychology, University of Oslo, Norway

[3] Centre for Fertility and Health, Norwegian Institute of Public Health

[4] Department of Global Public Health and Primary Care, Faculty of Medicine, University of Bergen

[5] Institute of Clinical Medicine, Faculty of Medicine, University of Oslo, Norway.

*Corresponding author: Norwegian Centre for Violence and Traumatic Stress Studies, Post box 181 Nydalen, 0409 Oslo, Norway. Phone: + 47 92 63 06 34. Email: m.t.g.dale@nkvts.no

Words 3.690

Keywords registry data; sick leave; workplace terror; psychosocial work environment; social support

For peer review only

ABSTRACT

Objectives

Studies show that social support may reduce the negative psychological effects of terror. The aim was to explore the effects of the psychosocial work environment on sick leave risk among governmental employees after a workplace bomb attack.

Design

We linked longitudinal survey data collected at 10 and 22 months after the bombing with registry data on doctor-certified sick leave collected from 42 months before the attack to 33 months after the attack. Odds ratios (ORs) and rate ratios (RRs) were estimated with mixed effects hurdle models.

Setting

The bombing of the government ministries in Oslo, Norway, 22 July 2011.

Participants

We identified 1625 participants from a cohort of 3520 employees working in the ministries during the bombing in 2011.

Results

After adjustment for confounders, social support from coworkers reduced the odds of sick leave (OR= 0.80, 95% CI 0.68-0.93), and there was marginal evidence for reduced odds with support from superior (OR= 0.87, 95% CI 0.87-1.03). A social work climate, an innovative climate, and a human resource primacy climate (HRP) reduced the sick leave risk (for example, HRP OR= 0.77, 95% CI 0.66-0.90). The hurdle model found no associations between psychosocial support at work and the duration of sick leave.

Conclusions

For peer review only - http://bmjopen.bmj.com/site/about/guidelines.xhtml

Psychosocial support at work can enhance employees' work ability after terror and reduce the sick leave risk by more than 20 percent. However, a supportive psychosocial work environment did not reduce the duration of sickness absence. The protective role of psychosocial work factors on sick leave may be most significant when employees are at work and interact with their work environment.

Strengths and limitations of this study

- This longitudinal study combines survey and registry data and examines the sick leave risk in a large population exposed to a workplace terror attack.
- This study has a relatively high response rate and an appropriate number of participants with and without sick leave, making it possible to compare the two samples.
- In terms of generalizability, the study sample consisted of a majority of highly educated government officials and bureaucrats.
- The use of self-reported data on psychosocial work environments may not objectively reflect available social support.
- A comprehensive approach that incorporates both intrinsic and extrinsic factors is needed to understand better how individuals cope in the aftermath of terror.

INTRODUCTION

When terrorism strikes the workplace, where people spend much of their time, survivors are highly affected.[1] The workplace serves as an essentially social context that provides routines, purpose, economic and social resources to one's life, all of which can be impaired after workplace violence.[2] After the Oslo bombing in 2011, terror-exposed individuals were at high risk of post-traumatic stress disorder (PTSD), depression and increased sick leave.[3 4] The magnitude of terror-related stressors might

For peer review only - http://bmjopen.bmj.com/site/about/guidelines.xhtml

have a different impact on survivors. Factors shown to affect the sickness absence are health, age, gender, coping style, personality, physical and psychosocial work factors, work schedule characteristics, and available social resources. [5-7] A common assumption is that the cumulative effects of negative life events can cause psychosocial morbidity, which is highly relevant after major disasters considering the many challenges that arise in the aftermath of such events.[8 9] Previous research has often focused on posttraumatic stress reactions and severity of psychopathology. However, many trauma-exposed individuals display high levels of resilience and coping in the wake of disaster, which is also apparent in prior studies by our research group. Specifically, a high proportion of government employees had a strong sense of attachment and commitment to the workplace, and some managed to work despite a very high symptom load.[3 10] In particular, it seems like the social network at work combined with a supportive organizational climate can be a significant source of support for employees.

Social support and good social relations affect health and act as protective resources against the negative impact of major life events.[11 12] Social support involves instrumental (e.g., material and financial), informational (e.g., advice and guidance), and emotional (e.g., empathy, trust and emotional venting) resources.[13] Previous studies show that these three supportive components can be directly associated with overall lower levels of psychological distress, independent of exposure to trauma.[14] Additionally, social support can act as a buffer by reducing or taking away the impact of a major trauma.[15] In line with this, studies show that after a natural disaster, low levels of social support have been associated to higher levels of PTSD and depression.[16-18] Further, when experiencing intense stress from terror, such as the September 11 terrorist attacks, research indicates that turning to others for help may prevent the development of long-lasting psychological sequelae.[19] Although the social network at work is considered to be more formal than social relations to family and friends, research

For peer review only - http://bmjopen.bmj.com/site/about/guidelines.xhtml

shows a strong association between support from colleagues and superiors and reduced sick leave.[20-22] However, the vast majority of studies on the effect of the psychosocial work environment on sick leave risk have not focused on workers exposed to a terror attack.

At present, it is unclear how the psychosocial work environment influences the sick leave risk and the duration of sick leave after terror. We assume that a high degree of social support from leaders and colleagues, combined with a supportive organizational climate with a concern for human resources, are essential for employees struggling with returning to work after unexpected and threatening workplace violence. Our study sample shared an extraordinary experience and psychosocial support at work may be even more critical for reestablishing routines and recovery of trust and safety. The present study aims to explore whether increasing levels of psychosocial support are associated with a corresponding decrease in employees' sick leave risk and reduction in sick leave days and consequently add knowledge to the limited literature on this association.

METHODS

Study population and data sources

This prospective cohort study includes web-based survey-data combined with registry data on doctor certified sick leave from ministerial employees in 14 of 17 Ministries, after a car bomb attack at the Norwegian government offices in Oslo, July 22nd, 2011. The terror bombing caused substantial damage to buildings and infrastructure, killing eight and injuring 209 people. Negative health reactions were prevalent among all employees, and the present study includes all, whether indirectly or directly, exposed to the bomb. 10.5 % of the employees were classified as directly exposed as they reported to be in the government district during the bomb explosion.[4]

For peer review only - http://bmjopen.bmj.com/site/about/guidelines.xhtml

The Norwegian Centre for Violence and Traumatic Stress Studies conducted the survey in collaboration with the National Institute of Occupational Health in Norway 10 and 22 months after the terrorist attack. Eligible participants were informed about the study through their ministries and received an invitation letter containing an unique log-in code to access the Web-based survey, including information on withdrawal procedures. Data on doctor certified sick leave was obtained from Statistics Norway and the Norwegian Labour and Welfare Administration.

For the purpose of this study, we used survey data on employees' background variables and information on the psychosocial work environment 10 months after the attack (T1) and 22 months after the attack (T2). The survey data was linked with registry data on doctor certified sick leave from a period of 42 months before the attack to 33 months after the attack. However, as the survey data was collected over a period of four months (about 8-12 months and 20-22 after the attack), we used registry data on sick leave from 13 to 21 months after the first survey (T1) and from 25 to 33 months after the second wave of the survey (T2). See Figure 1 *Timeline for sick leave outcomes and survey measures on the psychosocial work environment* for an overview of the timeline.

-Figure 1 about here-

All employees provided informed consent, and strict procedures were followed to ensure confidentiality. Willing participants received a postal invitation letter containing information about the study and withdrawal procedures. In the study's invitation letter, each employee was assigned an unique project identification ID-number and a log in code to access the study's Web-based questionnaire. Once the participants logged in to the Web-based questionnaire, they were informed that filling out the questionnaire was equivalent to a written consent of participation in the study. Further, they had to thick off "yes" to the question of linking survey data to registry data on doctor

For peer review only - http://bmjopen.bmj.com/site/about/guidelines.xhtml

certified sick leave. Based on the personal identification number from the Norwegian Population Register, Statistics Norway performed data linkage and de-identification.

Invited participants were ministerial employees who were employed in the Norwegian ministries at the time of the attack. Three of the initial 17 ministries did not agree to the study's consent procedure (two ministries; n = 440), or the office was located approximately one kilometer away from the government district with a significant proportion of the workforce based abroad (one ministry; n = 856). 3520 invited employees consented to participate; 59 could not be reached with information about the study and 482 employees left the ministerial job or changed ministry affiliation prior to study completion. The survey response rate was 56 % (1956 of 3520), where 1023 employees did not participate in the survey at any timepoint (at T1 and T2), while 1061 participated at both T1 and T2. In this study population, we identified 1625 participants eligible for the study, all with relevant data on the psychosocial work environment at T1 and/or T2 and with registry data for the 9- months following T1 and T2. Further details on design and participants have previously been described in a recent article from our research group.[3] See Figure 2 *Flow-chart displaying participant disposition* for more information on participants eligible for this study.

-Figure 2 about here-

Patient and public involvement

There is no direct patient involvement in this study. Neither patients nor the public was directly involved in our research's design, conduct, reporting, or dissemination plans.

Psychosocial work exposures

Social support at work (comprising two subscales) and supportive organizational culture (comprising three subscales) were measured by the General Nordic Questionnaire for Psychological and Social

For peer review only - http://bmjopen.bmj.com/site/about/guidelines.xhtml

Factors at Work, QPSNordic.[23] All responses were scored on an ordinal five-point scale ranging from "1 = very seldom or never" to" 5 = very often or always", and missing response to one of the items comprising each scale was allowed when computing a mean sumscore.

Social support from coworkers was measured with 2 items, and a typical item was "If needed, can you get support and help with your work from your coworkers?" Social support from superior comprised 3 items. A typical item was, "If needed, is your immediate superior willing to listen to your work-related problems?" Three subscales measured organizational culture: social climate, human resource primacy climate (HRP), and innovative climate, all comprising 3 items. An item representing social organizational climate was "Is the climate encouraging and supportive in your work unit?" A typical item measuring HRP was "Are workers well taken care of in your organization?" To measure innovative climate a relevant item was "Are workers encouraged to think of ways to do things better at your workplace?" Reliability tests of the five subscales have demonstrated approvable internal consistency measured with Chronbach's alpha from .71 to .83 and test-retest reliability from .72 to .83 with more than five weeks interval.[24]

Sick leave

The outcome of this study, doctor-certified sickness absence, was based on registry data on employment from Statistics Norway and registry data on sick leave from the Norwegian Labour and Welfare Administration. The former registry contains the number of expected workdays per quarter for a given person based on the person's contract(s) of employment. Weekends, public holidays and days of vacation are not considered potential workdays. For a person with full-time employment, there are roughly 170 expected workdays in each 9-month period explored in the study. The registry on sick leave contains the number of days a given person was absent from work per quarter due to doctor-

For peer review only

certified sick leave. The registry takes account of whether the person works full-time or part-time, and whether sick leave was graded or not (e.g. a person may be on 50% sick leave). For example, if a person with 80% employment (i.e., four expected workdays per week) gets one week of 50% doctor-certified sick leave in a quarter, the person will have 2 registered days of sickness absence for that quarter. Sick leave prior to the attack (from the first quarter of 2008 until the second quarter of 2011) was included as a potential confounder and defined as total sickness absence days divided by the number of expected work-days registered for this period. When used as an outcome variable in the main analysis, sick leave was examined in the two nine-month periods following the survey points at T1 and T2 (see Figure 1 for time frames).

Covariates

Covariates that could potentially influence the association between the psychosocial work environment and sick leave risk were considered a priori as potential confounders. The following covariates were included in the main analysis: time measured by the two survey waves (T1 contrasted with T2), sex, age, education, direct versus indirect exposure to terror, and sick leave prior to the attack. Proximity to the bomb explosion was assessed by asking employees where they were located during the explosion. Participants were given five alternative responses: 1) in the government district; 2) in downtown Oslo, but not in the government district; 3) in Oslo, but not downtown; 4) in Norway, but not in Oslo; 5) abroad. Only employees in the government district when the bomb exploded were defined as directly exposed to terror.

Analytic strategy

For peer review only - http://bmjopen.bmj.com/site/about/guidelines.xhtml

Mixed effects hurdle models were used in analyses, and participants contributed with data as long as they had at least one time point without missing values. As a result, the participants were included in the analysis as long as they had answered all variables used in the model for at least one survey wave (T1 and/or T2).

The frequency of sick leave was expressed as count data, with an excess of zeros (no sick leave).[25] As the count data was over-dispersed with variance larger than the mean, we used negative binomial hurdle models to estimate the incidence rate of sick leave.[26 27] Hurdle models are two-part models. The first part uses a logistic regression model to estimate the odds of the outcome being above zero vs. zero for various predictor levels in the model, summarized as odds ratios (ORs) compared to a set reference. In the present study, we estimated the odds ratio of sick leave among employees according to various psychosocial work environments, using crude (OR) and adjusted (aOR) odds ratios with 95% confidence intervals. The ORs compare the odds of having at least one day of sickness absence vs. no sickness absence for various predictor levels. The second part of the hurdle model studies the mean number of days of sickness absence, conditional on having at least one day of absence. For this part, it uses a truncated negative binomial regression model, where zero has been excluded for the positive counts.[28] By fitting the negative binomial model, we obtain mean count ratios, or rate ratios (RR, with 95% CIs). The rate ratio compares the mean number of days of sickness absence over levels of various predictors, including the main exposure. Consequently, the RRs estimate the exposure effect on the number of sickness absence days among those with at least one day of absence.

To account for varying person-time at risk (i.e. not all employees worked full-time during the observation period), the hurdle models were offset by employees' expected workdays in the relevant periods. Since data were collected longitudinally, with repeated measurements on individuals, we used a mixed effects extension of the hurdle model, with a random intercept on individuals. The effects of

For peer review only - http://bmjopen.bmj.com/site/about/guidelines.xhtml

social work factors were modeled in five separate hurdle regression analyses. Each work factor was included as a continuous variable, with raw mean scores, in the model.

The binomial regression analyses and the zero-truncated negative binominal analysis were computed in STATA version 15 (STATA Corporation, College Station, TX, USA) and R, using the R package GLMMadaptive.

RESULTS

Characteristics of the Study Population

In the study population of 1,625 employees, the prevalence of sick leave from 13 to 21 months after the attack was 25% (421 of 1626) after excluding employees leaving their job or changing ministry affiliation prior to study completion. After the second wave of the survey (T2), in the period from 25 months to 33 months after the attack, the sick leave prevalence was 24% (394 of 1625). Further, the mean number of sickness absence days was about 25 days at 13 to 21 months after the first survey (T1) and decreased to 22 days in the period of 25 to 33 months after the second wave of the survey (T2) among employees with sick leave. When expressed as the overall percentage among all employees in this study, this corresponds to 4.3 and 3.8 percent sick leave of all working days.

Table 1 *Characteristics in cases with no sick leave and those with sick leave* shows the distribution of background variables for sex, age, educational level, relationship status, directly- and indirectly exposed, psychosocial work environment, and sick leave, all retrieved from the questionnaire survey completed 10 months (T1) and 22 months (T2) after the terror attack.

-Table 1 about here-

For peer review only - http://bmjopen.bmj.com/site/about/guidelines.xhtml

Table 1 compares participants with and without sick leave 10 months after the attack (T1) and 22 months after the attack (T2). At both measurement points (T1 and T2) the group with sick leave had overall lower levels of education ($p < .01$) and higher percentage of females (71%) compared to the group without sick leave. At T1 there were more participants directly exposed to the bomb explosion among those with sick leave (14%) than those without sick leave (10%). Further, at both measurement points (T1 and T2) the group with sick leave reported overall lower scores in social support from coworkers ($p < .01$), social organizational climate (T1: $p = .02$; T2: $p < .01$), and human resource primacy climate ($p < .01$). At T1 the group with sick leave reported lower scores in support from leaders ($p < .01$), and at T2 those with sick leave reported lower scores in innovative organizational climate ($p = .02$). The groups with sick leave did not differ significantly from participants without sick leave with regard to marital status and age at T1 and T2.

Psychosocial support at work and sick leave risk

After adjusting for confounders, we observed that high support from coworkers was associated with overall reduced odds of sick leave for all time periods (aOR = 0.80, 95% CI 0.68-0.93). Similarly, support from superiors indicates reduced odds of sick leave (aOR = 0.87, 95% CI 0.79-1.01), but did not reach significance in the model (p=.063). A work environment with a social, innovative and human resource primacy climate reduced the odds of sick leave, where the estimates for all these three organizational climate variables are very similar with OR around 0.80. (E.g. HRP: OR=0.77, 95% CI 0.66-0.90). Moreover, the hurdle model estimated the duration of sick leave (RR) among those with sick leave. We found no significant associations between social support from superiors and coworkers and the duration of sickness absence among those with sick leave, nor between a supportive HRP, social climate and innovative organizational culture and the duration of sickness absence among those with sick leave

For peer review only - http://bmjopen.bmj.com/site/about/guidelines.xhtml

(see Table 2 *Two-part hurdle mixed effects models on work factors*). The RR-estimates indicate that all five factors measuring a supportive psychosocial work environment had no substantial impact on the duration of sick leave. Further, the overall effects of Time (T1 to T2) on sick leave scores after the attack were not significant (e.g. OR = 0.94, 95% CI 0.76-1.18 and RR = 0.85, 95% CI 0.71-1.02), indicating unchanged odds and rate ratio for sick leave at T2 when compared to T1 (the estimates for confounders are not shown in Table 2).

-Table 2 about here-

DISCUSSION

This is the first cohort study based on registry data on sick leave exploring longitudinally whether a supportive psychosocial work environment reduces the risk and duration of sickness absence after a terrorist attack on the workplace. Our findings indicate that a supportive psychosocial work environment can reduce the odds for sick leave by more than 20 percent. For the second part of the Hurdle-analysis, we observed no associations between a supportive psychosocial work environment and the duration of sickness absence. Further, we found that the proportion of women were higher among employees with sick leave when compared to those without sick leave.

Our first finding is in accordance with other studies (without focus on terrorism exposure) that find a protective effect of social support from the work environment, leading to a reduced risk of sick leave.[5-7] Especially, colleague support and appropriate supervision from the leader are important.[29 30] Moreover, previous findings show that high levels of social support at work are associated with reduced psychological distress, depression, and increased well-being.[12 14-16] Evidence for the protective effect of social support on mental health and distress is relevant for our outcome on sick leave risk, as employment and sick leave frequency are markers of functional recovery from trauma.[2] Most people

For peer review only - http://bmjopen.bmj.com/site/about/guidelines.xhtml

affected by disasters do not develop severe psychiatric disorders, though almost everyone with exposure to disaster trauma will experience distress for at least a brief period. A measure of sickness absence can capture individuals with sub-diagnostic distress after terrorism.

Our second main finding showed that psychosocial support was not associated with a reduction in the duration of sickness absence, indicating that the protective role of a supportive psychosocial work environment was significant only when employees managed to stay at work. This can partly be explained by studies showing that the relationship between social support at work and sickness absence is bidirectional or reciprocal.[20 31] A study by Sieurin et al (2009) found that long-term absentees often reported that their absence negatively affected their sense of belonging to the social workgroup.[32] One speculation is that sickness absence may affect the social relationships at work, and thereby adding to the challenges causing the sickness absence in the first place. Another factor could simply be that employees absent from work lose essential interaction with their work environment, and consequently, the positive effects of psychosocial support are reduced. Further, there might have been a "threshold effect" in our sample, where employees with sick leave had reached a higher level of adverse health and distress, where coping strategies and support from the work environment no longer have an effect on the frequency of sickness absence days.

The utility of social resources depends on the survivors' ability to seek and receive support from their social and interpersonal resources in the aftermath of a terror attack.[16] Clearly, employees with sick leave had significantly more psychological strains and symptoms compared to those without sick leave, and they were slightly older with proportionally more women, which is in line with previous findings on sick leave risk with evidence for gender, older age, and previous history of sickness as risk factors.[19 33-35] According to the social selection hypothesis, people struggling with post-traumatic stress symptoms such as withdrawal, depression, and irritability may not seek necessary social support but

For peer review only - http://bmjopen.bmj.com/site/about/guidelines.xhtml

instead, diminish their interpersonal relations over time.[14] Further, employees experiencing psychological distress might evaluate the psychosocial work environment more negatively, affecting the subjective appraisals of leader and coworker support. This explanation can be related to a tendency in our results where leader support was not the most essential protective factor against sick leave for the employees after the terror attack. It could be that employees suffering from psychological distress after the attack perceived their immediate leader to be less supportive, as distress could have a negative effect on subjective appraisals of leader support. [36] However, the causation could as well be reversed, where employees with a poor psychosocial work environment experience more psychological distress.

Strengths and limitations

By means of an unique longitudinal design with registry data on sick leave, we were able to extend our knowledge of sick leave risk, and how it relates to the psychosocial work environment after terror. Existing research on terrorism and health effects are mostly focused on psychotraumatology and mental disorders such as PTSD, and sick leave may be a better objective measure of general functioning and health, especially consistently and routinely collected doctor certified data. [6 37]

The study has several limitations. First, the study population consisted of a majority of highly educated government officials and bureaucrats and our findings may not necessarily be generalized to other populations. [38] Recent results from the same cohort study found that the sick leave rates for ministerial employees were lower than in the general Norwegian population prior to, as well after, the bomb explosion.[3] Furthermore, it can be that the terror attack could have greater negative health effects in another sample, as the sick leave rates was higher for example among Norwegian tourists exposed to the South-East Asian tsunami in 2004.[39] Second, we used data on doctor-certified sick leave.

For peer review only - http://bmjopen.bmj.com/site/about/guidelines.xhtml

In Norway, the workers are entitled to be home from work up to three consecutive days, four times per year, without doctor certification. Further, if the employer had signed the Agreement for a More Inclusive Working Life (IA Agreement), the employee could report sick leave for 8 consecutive calendar days without doctor certification, up to a total of 24 days per year, without limiting the number of times. We assume that many governmental employees were covered by the IA Agreement during the follow-up period. Therefore, the difference between registered and actual sick leave may be larger than anticipated, measured as incidence as well as days. Therefore, the overall incidence rate of sick leave would be higher if this was included in the official registry. Third, it should be noted that sickness absence is not only indicative of health problems, but strongly affected by factors such as education, health behaviours and sick pay insurance.[40] As such, sickness absence is not necessarily a precise measure of the health difficulties in the aftermath of a terrorist attack.

Fourth, the assessment of the psychosocial work environment was based on self-reported measures and may be influenced by social desirability, underreporting, recall bias, and other response bias, where subjective appraisals of the work environment are closely linked to personality traits and negative affect.[41] However, the QPS_{Nordic} has been validated as an effective instrument for measuring psychological and social factors at work and should be relatively insensitive to personality and emotions, for example respondents were asked about how frequently a situation occurs instead of degrees of agreement or satisfaction.[22] Finally, we had no information on workplace social support prior to the terrorist attack, allowing a comparison of the effect of social support on sick leave before and after the terrorist attack. However, we had data on sick leave before the attack and were able to adjust for important confounders, such as previous sickness absence in the main analysis, a factor that could potentially influence later sick leave risk, and participant ratings of the psychosocial work environment.

For peer review only - http://bmjopen.bmj.com/site/about/guidelines.xhtml

Implications

It seems reasonable to conclude that psychosocial support at work can reduce the sick leave risk after terror. The workplace should mobilize its social and supportive resources before employees become sick-listed, and before potentially new negative psychological and physical reactions have time to develop and at worse result in long-term sick leave. Especially as the psychosocial work factors appeared to have no major impact on the duration of sickness absence. Future research should investigate whether the associations are causal, and whether these findings can be replicated in other populations, and over a longer time period. A more comprehensive approach that incorporates both intrinsic and extrinsic factors is needed to better capture and understand how individuals cope in the aftermath of terror and to identify the difficulties for return to work.

Acknowledgements We thank all the governmental employees who took part in this study.

Author contributions TH designed the study. MTGD and AFN analyzed the data and MTGD drafted the paper. All authors, TH, MB, HG, AFN and MTGD, participated in project meetings where the analysis plan and data interpretation were discussed and where the article was critically revised.

Funding This work was funded by grants from Research Council Norway, grant number 227039.

Conflict of interests None declared.

Patient consent for publication Not required.

For peer review only - http://bmjopen.bmj.com/site/about/guidelines.xhtml

Ethical approval Informed consent was obtained from all participants, and the Regional Committee for Ethics in Medical Research approved the study (reference number: 2011/1577).

Provenance and peer review Not commissioned; externally peer reviewed.

Data sharing statement Data are available from the corresponding author on reasonable request. The datasets generated during and/or analyzed during the current study are not publicly available due to confidentiality agreements made with participants.

ORCID iD Maria Teresa Grønning Dale https://orcid.org/0000-0002-0972-2996

REFERENCES

1. North CS, Pfefferbaum B, Hong BA, et al. The Business of Healing: Focus Group Discussions of Readjustment to the Post-9/11 Work Environment Among Employees of Affected Agencies. *Journal of Occupational and Environmental Medicine* 2010;52(7):713-18. doi: 10.1097/JOM.0b013e3181e48b01
2. Weiseth L, Heir T. Workplacce and organizational disasters: response and planning. In: Ursano R ea, ed. Textbook of disaster psychiatry. Cambridge: Cambridge University Press 2017:261-69.
3. Hansen MB, Berthelsen M, Nissen A, et al. Sick leave before and after a work-place targeted terror attack. *International archives of occupational and environmental health* 2019;92(3):327-35. doi: 10.1007/s00420-018-1390-8
4. Hansen MB, Birkeland MS, Nissen A, et al. Prevalence and Course of Symptom-Defined PTSD in Individuals Directly or Indirectly Exposed to Terror: A Longitudinal Study. *Psychiatry* 2017;80(2):171-83. doi: 10.1080/00332747.2016.1230983
5. Boot CRL, van Drongelen A, Wolbers I, et al. Prediction of long-term and frequent sickness absence using company data. *Occupational Medicine* 2017;67(3):176-81. doi: 10.1093/occmed/kqx014
6. Melchior M, Niedhammer I, Berkman LF, et al. Do psychosocial work factors and social relations exert independent effects on sickness absence? A six year prospective study of the GAZEL cohort. *Journal of Epidemiology and Community Health* 2003;57(4):285-93. doi: 10.1136/jech.57.4.285
7. Michie S, Williams S. Reducing work related psychological ill health and sickness absence: a systematic literature review. *Occup Environ Med* 2003;60(1):3-9. doi: 10.1136/oem.60.1.3

For peer review only - http://bmjopen.bmj.com/site/about/guidelines.xhtml

8. Arnberg FK, Gudmundsdóttir R, Butwicka A, et al. Psychiatric disorders and suicide attempts in Swedish survivors of the 2004 southeast Asia tsunami: a 5 year matched cohort study. *The Lancet Psychiatry* 2015;2(9):817-24. doi: 10.1016/S2215-0366(15)00124-8
9. Bonanno GA, Brewin CR, Kaniasty K, et al. Weighing the Costs of Disaster:Consequences, Risks, and Resilience in Individuals, Families, and Communities. *Psychological Science in the Public Interest* 2010;11(1):1-49. doi: 10.1177/1529100610387086
10. Dale MTG, Nissen A, Berthelsen M, et al. Post-traumatic stress reactions and doctor-certified sick leave after a workplace terrorist attack: Norwegian cohort study. *BMJ Open* 2020;10(2):e032693. doi: 10.1136/bmjopen-2019-032693
11. Diener E, Seligman MEP. Very Happy People. *Psychological Science* 2002;13(1):81-84. doi: 10.1111/1467-9280.00415
12. Taylor SE. Social support: A review. The Oxford handbook of health psychology. New York, NY, US: Oxford University Press 2011:189-214.
13. Cohen S. Social Relationships and Health. Am Psychol. 2004;59(8):676-684. doi: 10.1037/0003-066X.59.8.676.
14. Santini ZI, Koyanagi A, Tyrovolas S, et al. The association between social relationships and depression: A systematic review. *Journal of Affective Disorders* 2015;175:53-65. doi: 10.1016/j.jad.2014.12.049
15. Cohen S, Wills TA. Stress, social support, and the buffering hypothesis. *Psychol Bull* 1985;98(2):310-57.
16. Arnberg FK, Hultman CM, Michel P-O, et al. Social Support Moderates Posttraumatic Stress and General Distress After Disaster. *Journal of Traumatic Stress* 2012;25(6):721-27. doi: 10.1002/jts.21758
17. Fullerton CS, Ursano RJ, Kao T-C, et al. Diaster-realted bereavement: Acute symptoms and subsequent depression. *Aviation, Space, and Environmental Medicine* 1999;70(9):902-09.
18. Madakasira S, O'Brien KF. Acute posttraumatic stress disorder in victims of a natural disaster. *Journal of Nervous and Mental Disease* 1987;175(5):286-90. doi: 10.1097/00005053-198705000-00008
19. Galea S, Ahern J, Resnick H, et al. Psychological Sequelae of the September 11 Terrorist Attacks in New York City. *New England Journal of Medicine* 2002;346(13):982-87. doi: 10.1056/NEJMsa013404
20. Knapstad M, Holmgren K, Hensing G, et al. Previous sickness absence and current low perceived social support at work among employees in the general population: a historical cohort study. *BMJ open* 2014;4(10):e005963. doi: 10.1136/bmjopen-2014-005963
21. Vahtera J, Kivimäki M, Pentti J, et al. Effect of change in the psychosocial work environment on sickness absence: a seven year follow up of initially healthy employees. *Journal of epidemiology and community health* 2000;54(7):484-93. doi: 10.1136/jech.54.7.484
22. Moreau M, Valente F, Mak R, et al. Occupational stress and incidence of sick leave in the Belgian workforce: the Belstress study. *Journal of Epidemiology and Community Health* 2004;58(6):507-16. doi: 10.1136/jech.2003.007518
23. Dallner M EA-L, Gamberale F, Hottinen V, Knahrdahl S, Lindtröm K , et al Validation of the General Nordic Questionnaire (QPSNordic) for psychological and social factors at work. *Copenhagen: Nordic Council of Ministers, 2000*;NORD 2000:12
24. Lindström K, Ørhede E, Elo A-L, et al. User's guide for the QPSNordic : General Nordic Questionnaire for Psychological and Social Factors at Work. København: Nordic Council of Ministers 2000.
25. Cameron AC, Trivedi PK. Regression analysis of count data. Second ed. Cambridge, UK: Cambridge University Press 2013.

For peer review only - http://bmjopen.bmj.com/site/about/guidelines.xhtml

26. Cheung YB. Zero-inflated models for regression analysis of count data: a study of growth and development. *Statistics in Medicine* 2002;21(10):1461-69. doi: 10.1002/sim.1088
27. Zeileis A. KC, Jackman S. Regression Models for Count Data in R. *Journal of Statistical Software* 2008;27(8) doi: 10.18637/jss.v027.i08
28. Rodriguez G. Models for count data with overdispersion. Retrieved from Princeton University: 2013, November 6th. http://dataprincetonedu/wws509/notes/c4apdf
29. Finne LB, Christensen JO, Knardahl S. Psychological and social work factors as predictors of mental distress: a prospective study. *PloS one* 2014;9(7):e102514-e14. doi: 10.1371/journal.pone.0102514
30. Kuoppala J, Lamminpää A, Liira J, et al. Leadership, Job Well-Being, and Health Effects—A Systematic Review and a Meta-Analysis. *Journal of Occupational and Environmental Medicine* 2008;50(8):904-15. doi: 10.1097/JOM.0b013e31817e918d
31. Hausser JA, Mojzisch A, Niesel M, et al. Ten years on: A review of recent research on the Job Demand-Control (-Support) model and psychological well-being. *Work & Stress* 2010;24(1):1-35. doi: 10.1080/02678371003683747
32. Sieurin L, Josephson M, Vingård E. Positive and negative consequences of sick leave for the individual, with special focus on part-time sick leave. *Scandinavian Journal of Public Health* 2008;37(1):50-56. doi: 10.1177/1403494808097171
33. Dekkers-Sánchez PM, Hoving JL, Sluiter JK, et al. Factors associated with long-term sick leave in sick-listed employees: a systematic review. *Journal of Occupational and Environmental Medicine* 2008;65(3):153-57. doi: 10.1136/oem.2007.034983
34. Mastekaasa A. The gender gap in sickness absence: long-term trends in eight European countries. *European Journal of Public Health* 2014;24(4):656-62. doi: 10.1093/eurpub/cku075
35. North CS, Nixon SJ, Shariat S, et al. Psychiatric Disorders Among Survivors of the Oklahoma City Bombing. *JAMA* 1999;282(8):755-62. doi: 10.1001/jama.282.8.755
36. Birkeland MS, Nielsen MB, Hansen MB, et al. The impact of a workplace terrorist attack on employees' perceptions of leadership: A longitudinal study from pre- to postdisaster. *The Leadership Quarterly* 2017;28(5):659-71. doi: 10.1016/j.leaqua.2017.01.002
37. Kivimäki M, Head J, Ferrie JE, et al. Sickness absence as a global measure of health: evidence from mortality in the Whitehall II prospective cohort study. *BMJ* 2003;327(7411):364. doi: 10.1136/bmj.327.7411.364
38. Robroek SJW, van Lenthe FJ, Burdorf A. The role of lifestyle, health, and work in educational inequalities in sick leave and productivity loss at work. *International archives of occupational and environmental health* 2013;86(6):619-27. doi: 10.1007/s00420-012-0793-1
39. Heir T, Piatigorsky A, Weisæth L. Posttraumatic stress symptom clusters associations with psychopathology and functional impairment. *Journal of Anxiety Disorders* 2010;24(8):936-40. doi: 10.1016/j.janxdis.2010.06.020
40. Palme M, Persson M. Sick pay insurance and sickness absence: Some europeean cross-country observations and a review of previous reseach. *Journal of Economic Surveys* 2020;34(1):85-108. doi: 10.1111/joes.12351
41. Wainwright D, Calnan M. Work stress: the making of a modern epidemic. Buckingham: Open University Press, 2002.

For peer review only - http://bmjopen.bmj.com/site/about/guidelines.xhtml

Table 1 Characteristics in cases with no sick leave and those with sick leave at 10 months (T1) and 22 months (T2) after the 2011 terrorist attack in the cohort with 1,625 Norwegian ministerial employees.

	T1				T2			
**Characteristics**	**No sick leave n = 1023**	**Sick leave n = 349**	**χ^2/F**	**p[a]**	**No sick leave n= 990**	**Sick leave n= 324**	**χ^2/F**	**p[a]**
Females, n (%)	528 (51.6)	248 (71.1)*	40.1	<0.01	525 (53.0)	229 (70.7)*	31.1	<0.01
Education, n (%)			41.7	<0.01			38.4	<0.01
< 13 years	82 (8.0)	68 (19.5)*			92 (9.4)	72 (22.2)*		
13-16 years	224 (21.9)	89 (25.5)*			218 (22.1)	73 (22.5)		
> 16 years	717 (70.1)	192 (55.0)*			677 (68.5)	179 (55.3)*		
Married/Cohabiting, n (%)	598 (75.5)	183 (68.3)	5.4	0.02	730 (73.7)	245 (75.9)	0.6	0.45
Age (M ± SD)	46.3 ± 10.6	47.3 ± 10.5	2.3	0.14	46.4 ± 10.4	47.6 ± 10.7	3.2	0.07
Directly exposed, n (%)	100 (9.8)	49 (14.0)*	4.9	0.03	112 (11.4)	32 (9.9)	0.5	0.47
Sick-leave days (M ± SD) [b]		24.8 ± 34.6				20.8 ± 25.7		
Support from superior, score 1-5 (M ± SD)	4.0 ± 0.8	3.8 ± 0.9*	11.5	<0.01	4.0 ± 0.9	3.9 ± 0.8	3.7	0.06
Support from coworkers, score 1-5 (M ± SD)	4.1 ± 0.7	3.9 ± 0.8*	8.6	<0.01	4.1 ± 0.7	4.0 ± 0.8*	7.4	<0.01
Social organizational climate, score 1-5 (M ± SD)	3.9 ± 0.7	3.8 ± 0.7*	5.4	0.02	3.9 ± 0.7	3.8 ± 0.7*	10.2	<0.01
Human resource primacy climate score 1-5 (M ± SD)	3.4 ± 0.7	3.2 ± 0.8*	18.5	<0.01	3.4 ± 0.7	3.2 ± 0.8*	7.3	<0.01
Innovative organizational climate score 1-5 (M ± SD)	3.6 ± 0.7	3.5 ± 0.8	2.9	0.08	3.6 ± 0.7	3.5 ± 0.7*	5.4	0.02

Table showing case numbers and within-group percentages
*Differs significantly from those without sick leave
[a] P values were calculated using ANOVA for continuous variables and χ^2 test for categorical variables.
[b] Nine-months period following the survey questionnaires at T1 and T2
M Mean; SD Standard Deviation

For peer review only - http://bmjopen.bmj.com/site/about/guidelines.xhtml

Table 2 Two-part hurdle mixed effects models on work factors among 1,625 Norwegian ministerial employees measured 10 and 22 months after the terrorist attack 22nd of July, 2011, and risk of sick leave measured nine-month after each survey.

	Part I: Binary logistic model		Part II: Negative binomial count model	
**Variables**	**OR**	**OR[a]**	**RR**	**RR[a]**
**Psychosocial work environment**				
Social support/interactions:				
Support from superior	0.81 (0.70-0.93)*	0.87 (0.76-1.01)	0.90 (0.80-1.00)	0.94 (0.84-1.05)
Support from coworkers	0.75 (0.64-0.89)*	0.80 (0.68-0.93)*	0.89 (0.79-1.01)	0.94 (0.83-1.07)
Organizational culture:				
Social organizational climate	0.72 (0.59-0.86)*	0.78 (0.66-0.93)*	0.98 (0.85-1.12)	1.03 (0.90-1.18)
Human resource primacy climate	0.70 (0.59-0.83)*	0.77 (0.66-0.90)*	0.88 (0.77-1.00)	0.93 (0.82-1.05)
Innovative organizational climate	0.79 (0.66-0.95)*	0.82 (0.69-0.98)*	0.95 (0.84-1.09)	0.99 (0.86-1.13)

OR Odds Ratio [95% Confidence Interval (CI)] for sickness absence from work (Yes/No)

RR Rate Ratio (95% CI) for number of sickness absence days among those with sick leave

[a] adjusted for time (T1 contrasted with T2), sex, age, education, sick leave prior to attack and direct exposure to the bomb explosion.

[*] significant

For peer review only - http://bmjopen.bmj.com/site/about/guidelines.xhtml

Figure 1 Timeline for sick leave outcomes and survey measures on psychosocial work environment

- Uploaded separately in PDF format-

Figure 2 Flow-chart displaying participant disposition

- Uploaded separately in PDF format-

For peer review only

Figure 1 Timeline for sick leave outcomes and survey measures on psychosocial work environment

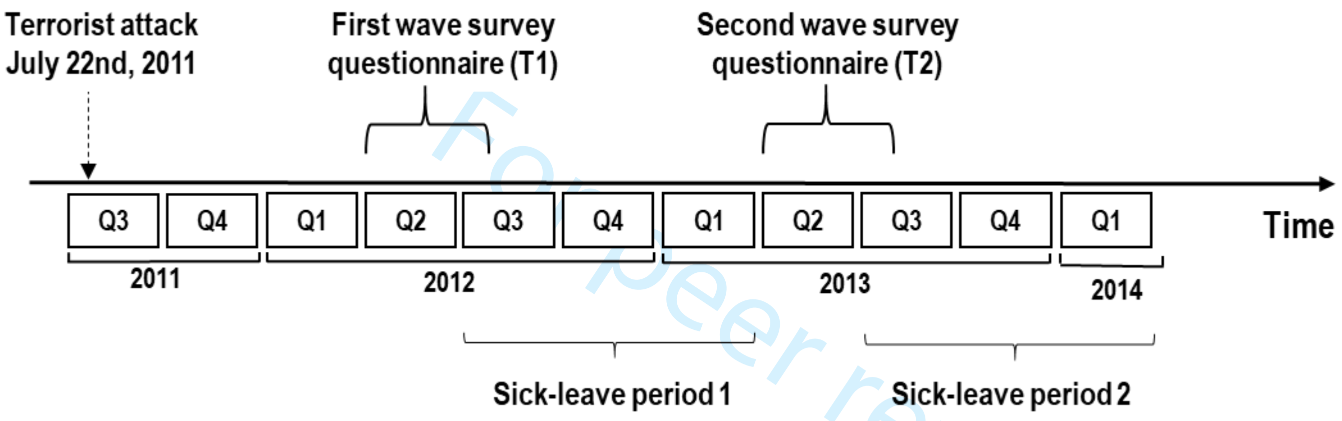

Q: Quarter
T1: Starts about 10 months after the attack
T2: Starts about 22 months after the attack

For peer review only

Figure 2 Flow-chart displaying participant disposition

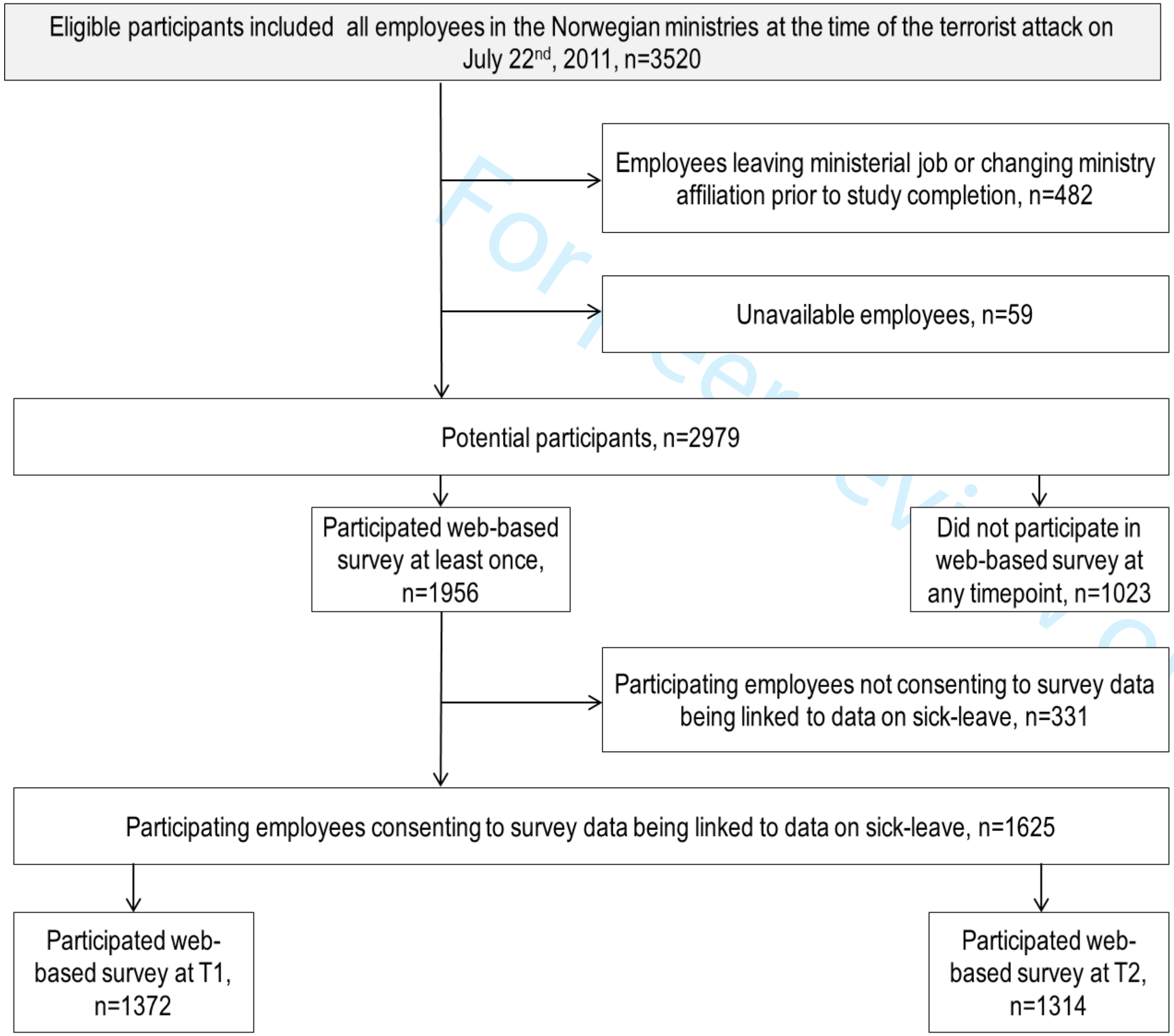

STROBE Statement—Checklist of items that should be included in reports of ***cohort studies***

	Item No	Recommendation	Page No
**Title and abstract**	1	(*a*) Indicate the study's design with a commonly used term in the title or the abstract	1
		(*b*) Provide in the abstract an informative and balanced summary of what was done and what was found	2
**Introduction**			
Background/rationale	2	Explain the scientific background and rationale for the investigation being reported	3-5
Objectives	3	State specific objectives, including any prespecified hypotheses	5
**Methods**			
Study design	4	Present key elements of study design early in the paper	5
Setting	5	Describe the setting, locations, and relevant dates, including periods of recruitment, exposure, follow-up, and data collection	5-6 Figure1
Participants	6	(*a*) Give the eligibility criteria, and the sources and methods of selection of participants. Describe methods of follow-up	7-6
		(*b*) For matched studies, give matching criteria and number of exposed and unexposed	n/a
Variables	7	Clearly define all outcomes, exposures, predictors, potential confounders, and effect modifiers. Give diagnostic criteria, if applicable	7-9
Data sources/ measurement	8*	For each variable of interest, give sources of data and details of methods of assessment (measurement). Describe comparability of assessment methods if there is more than one group	9-10
Bias	9	Describe any efforts to address potential sources of bias	9
Study size	10	Explain how the study size was arrived at	7 Figure 2
Quantitative variables	11	Explain how quantitative variables were handled in the analyses. If applicable, describe which groupings were chosen and why	7-8
Statistical methods	12	(*a*) Describe all statistical methods, including those used to control for confounding	7-9
		(*b*) Describe any methods used to examine subgroups and interactions	n/a (not relevant)
		(*c*) Explain how missing data were addressed	10
		(*d*) If applicable, explain how loss to follow-up was addressed	Figure 2
		(*e*) Describe any sensitivity analyses	n/a
**Results**			
Participants	13*	(a) Report numbers of individuals at each stage of study—eg numbers potentially eligible, examined for eligibility, confirmed eligible, included in the study, completing follow-up, and analysed	6-7 and 11-12
		(b) Give reasons for non-participation at each stage	Figure 2
		(c) Consider use of a flow diagram	
Descriptive data	14*	(a) Give characteristics of study participants (eg demographic, clinical, social) and information on exposures and potential confounders	11 Table 1
		(b) Indicate number of participants with missing data for each variable of interest	
		(c) Summarise follow-up time (eg, average and total amount)	

For peer review only - http://bmjopen.bmj.com/site/about/guidelines.xhtml

Outcome data	15*	Report numbers of outcome events or summary measures over time	Table 1

For peer review only

For peer review only - http://bmjopen.bmj.com/site/about/guidelines.xhtml

Main results	16	(*a*) Give unadjusted estimates and, if applicable, confounder-adjusted estimates and their precision (eg, 95% confidence interval). Make clear which confounders were adjusted for and why they were included<br>(*b*) Report category boundaries when continuous variables were categorized<br>(*c*) If relevant, consider translating estimates of relative risk into absolute risk for a meaningful time period	11-12<br>Table 2
Other analyses	17	Report other analyses done—eg analyses of subgroups and interactions, and sensitivity analyses	n/a
**Discussion**			
Key results	18	Summarise key results with reference to study objectives	13-15
Limitations	19	Discuss limitations of the study, taking into account sources of potential bias or imprecision. Discuss both direction and magnitude of any potential bias	15
Interpretation	20	Give a cautious overall interpretation of results considering objectives, limitations, multiplicity of analyses, results from similar studies, and other relevant evidence	17
Generalisability	21	Discuss the generalisability (external validity) of the study results	15-16
**Other information**			
Funding	22	Give the source of funding and the role of the funders for the present study and, if applicable, for the original study on which the present article is based	17

*Give information separately for exposed and unexposed groups.

Note: An Explanation and Elaboration article discusses each checklist item and gives methodological background and published examples of transparent reporting. The STROBE checklist is best used in conjunction with this article (freely available on the Web sites of PLoS Medicine at http://www.plosmedicine.org/, Annals of Internal Medicine at http://www.annals.org/, and Epidemiology at http://www.epidem.com/). Information on the STROBE Initiative is available at http://www.strobe-statement.org.

For peer review only
